# Isolation and Antibiofilm Activity of Bacteriophages against *Cutibacterium acnes* from Patients with Periprosthetic Joint Infection

**DOI:** 10.3390/v16101592

**Published:** 2024-10-10

**Authors:** Baixing Chen, Marco Chittò, Siyuan Tao, Jeroen Wagemans, Rob Lavigne, R. Geoff Richards, Willem-Jan Metsemakers, T. Fintan Moriarty

**Affiliations:** 1 Department of Trauma Surgery, University Hospitals Leuven, 3000 Leuven, Belgium; baixing.chen86@gmail.com (B.C.);; 2 Department of Development and Regeneration, KU Leuven, 3000 Leuven, Belgium; 3 AO Research Institute Davos, 7270 Davos, Switzerland; 4 Laboratory for Biointerfaces, Empa, 9014 St. Gallen, Switzerland; 5 Laboratory of Gene Technology, KU Leuven, 3000 Leuven, Belgium

**Keywords:** *Cutibacterium acnes*, bacteriophage, phage, peri-prosthetic joint infection, fracture-related infection, biofilm

## Abstract

Background: Infections following shoulder surgery, particularly periprosthetic joint infection (PJI), are challenging to treat. *Cutibacterium acnes* is the causative pathogen in 39% to 76% of these cases. This study explores the efficacy of bacteriophage therapy as an alternative to conventional antibiotics for treating such infections. Methods: Nine phages with lytic activity were isolated from the skin of humans using *C. acnes* ATCC 6919 as the indicator host. These phages were tested individually or in combination to assess host range and antibiofilm activity against clinical strains of *C. acnes* associated with PJIs. The phage cocktail was optimized for broad-spectrum activity and tested in vitro against biofilms formed on titanium discs to mimic the prosthetic environment. Results: The isolated phages displayed lytic activity against a range of *C. acnes* clinical isolates. The phage cocktail significantly reduced the bacterial load of *C. acnes* strains 183, 184, and GG2A, as compared with untreated controls (*p* < 0.05). Individual phages, particularly CaJIE7 and CaJIE3, also demonstrated significant reductions in bacterial load with respect to specific strains. Moreover, phages notably disrupted the biofilm structure and reduced biofilm biomass, confirming the potential of phage therapy in targeting biofilm-associated infections. Conclusions: Our preclinical findings support the potential of phage therapy as a viable adjunct to traditional antibiotics for treating *C. acnes* infections in orthopedic device-related infections. The ability of phages to disrupt biofilms may be particularly beneficial for managing infections associated with prosthetic implants.

## 1. Introduction

Infections following shoulder surgery, including periprosthetic joint infection (PJI) or fracture-related infection (FRI), can be a challenging complication [1,2]. The incidence of PJI after primary shoulder procedures ranges from 0.7% to 1.8%, and this increases to 4% and 15.4% for revision surgeries [3,4]. *Cutibacterium acnes* (formerly Propionibacterium) has emerged as the predominant pathogen in shoulder PJI, accounting for 39–76% of cases [5,6,7]. With respect to FRI, *C. acnes* has also been identified as an important causative pathogen, especially in patients without clinical signs of infection [8]. The high prevalence of *C. acnes* contrasts sharply with many other anatomical locations, where Staphylococcus aureus is the primary pathogen of concern. The prevalence of *C. acnes* in shoulder arthroplasty is believed to be due to the unique microbiome of the shoulder joint, characterized by a high density of sebaceous glands that provides an ideal environment for *C. acnes* colonization, particularly in lipid-rich areas [9]. Clinical experience proves that *C. acnes* shoulder infections can be challenging to treat, with some patients experiencing recurrent infections despite antibiotic therapy [10,11]. Considering that conventional antibiotic therapy has limited efficacy in *C. acnes* orthopedic device-related infections (ODRIs), alternative and complementary therapeutic strategies are required.

Bacteriophage (phage) therapy, which employs viruses to target and lyse specific bacteria, has reemerged as a potential therapy for ODRIs [12]. Although still emerging, evidence supporting the effectiveness of phage therapy in treating bone and joint infections is expanding, with several clinical cases demonstrating its potential to address infections resistant to conventional treatments [13]. Phages are particularly appealing for their high specificity, which targets only specific bacteria while leaving the rest of the microbiome intact, their self-replicating properties, their ability to lyse bacteria without contributing to the spread of antibiotic resistance, and their potential to disrupt bacterial biofilms. *C. acnes* phages are naturally present on the human skin [14]. The host range of isolated *C. acnes* phages is quite broad, with individual phages capable of targeting *C. acnes* isolates across multiple clades within the *C. acnes* population [15]. This relatively broad host range makes *C. acnes* phages ideal candidates for treating *C. acnes* infections. Studies focused on the therapeutic application of *C. acnes* phages showed that when *C. acnes*-induced skin inflammation was treated with phages, the inflammatory lesions significantly decreased [15,16,17].

Although phage therapy may be a valid therapeutic approach for *C. acnes*, no studies have yet investigated the efficacy of phages against *C. acnes* from deep infections and determined their efficacy in disrupting *C. acnes* biofilms. Our study aims to fill this gap by isolating and characterizing phages from the skin microbiota of healthy individuals that are effective against C. acnes, particularly strains implicated in PJI. We examine the lytic activity and antibiofilm activity of these phages against clinical isolates of *C. acnes*. Finally, a phage cocktail is identified that displays high efficacy against clinical isolates that are relevant in ODRIs.

## 2. Materials and Methods

### 2.1. Bacterial Strains and Culture Conditions

The reference strain of *C. acnes* (ATCC 6919) was purchased from the American Type Culture Collection (ATCC, Manassas, VA, USA). Nine clinical PJI isolates were cultured at the University Hospital Zurich (*C. acnes* 174, 182, 183, 184, 185, 186, 1104, 1105, and 1113), and five PJI clinical strains were isolated from Musgrave Park Hospital, Belfast, Northern Ireland (*C. acnes* GG2A, LED2, W1392, RB1B, and WBT1AA). Bacterial stock cultures were stored in 20% (*v*/*v*) glycerol at −80 °C. The *C. acnes* were anaerobically grown on brain heart infusion (BHI, Sigma-Aldrich, Steinheim, Germany) agar in a GasPak EZ System (BD Diagnostics, Allschwil, Switzerland) at 37 °C for three to five days.

### 2.2. Isolation and Propagation of Phages

*C. acnes* phages were isolated from nine healthy individuals. Sterile dry swabs were used to collect samples from skin in the alar crease, retroauricular crease, and occipital areas. The swabs were immediately placed into sterile tubes containing 5 mL of BHI broth and cultured in an anaerobic atmosphere at 37 °C for 72 h. Samples were subsequently centrifuged at 3220× *g* for 10 min, and the supernatants were filtered through a 0.45 µm filter and subsequently through a 0.22 µm filter (Millex, Merck Millipore, Cork, Ireland) to remove bacterial debris. *C. acnes* ATCC 6919 was used as a phage host for the isolation of phages from all swabs. Phages in the supernatant were identified by plaque formation using the spot assay method [18]. Briefly, 100 µL of the *C. acnes* ATCC 6919 culture was added to 4 mL of warm BHI soft agar (0.7%), mixed gently, and poured onto a BHI agar plate (1.5%), and the mixture was allowed to solidify at room temperature. Subsequently, 10 µL of the diluted filtrate was spotted on the top of the plate and incubated anaerobically to observe the clear regions generated as a result of phage lysing their host cells for 72 h. Single isolated plaques were picked to start a second round of amplification. The purification passages were repeated at least five times to ensure purity of the phage. The purified phages were stored in 1 mL of Dulbecco phosphate buffer saline (DPBS, [2.7 mM potassium chloride (KCl), 1.5 mM potassium phosphate monobasic (KH_2_PO_4_), 137.9 mM sodium chloride (NaCl), 8.1 mM sodium phosphate dibasic (Na_2_HPO_4_-12H_2_O)]) at 4 °C. To propagate these phages, the double-agar overlay method was used with some modifications. Using a sterile loop, the top agar layer (containing the phages) was carefully removed from the plate and transferred to a falcon tube with 10 mL of DPBS. The resuspended mixture was subsequently centrifuged at 3220× *g* for 10 min to pellet the agar and any bacterial debris, and the supernatants were filtered through a sterile 0.45 µm filter and subsequently through a sterile 0.22 µm filter to remove any remaining bacterial debris. Lastly, the resultant phage suspensions were stored at 4 °C. The plaque morphology was measured and analyzed by the software Scan^®^ 1200 (Interscience, Saint Nom, France).

### 2.3. Host Range Analysis

The host range of each phage was determined through a spot assay using the *C. acnes* isolates listed above. A bacterial lawn was prepared by mixing 100 µL of a fully grown bacterial culture (as mentioned in Section 2.1) with 4 mL of BHI soft agar (0.7%), pouring it over a BHI agar plate (1.5%) and letting it dry. A 10 µL aliquot of each phage suspension dilution (10^−1^ to 10^−9^) was spotted onto each bacterial overlay and anaerobically incubated at 37 °C for 72 h.

The susceptibility of bacteria to *C. acnes* was assessed using the efficacy of plating (EOP) method. EOP was determined by calculating the ratio of plaque forming units (PFUs) on the clinical strains being tested in comparison with the isolation/propagation host bacterium (ATCC 6919). EOP values exceeding 0.5 were classified as “high” efficiency, while values ranging from 0.2 to 0.5 were classified as “medium” efficiency, and values ranging between 0.001 and 0.2 were classified as “low” efficiency. A value of 0.0 indicated lack of any effectiveness against the target strain [19].

### 2.4. Phage Cocktail

A mixed phage suspension containing four different phages (Phages CaJIE1, CaJIE3, CaJIE7, and CaJIE8), referred to as the “phage cocktail”, was formulated to include phages selected for their ability to infect a wide variety of bacterial strains. This selection was chosen to ensure the broadest possible host range, enhancing the cocktail’s efficacy against diverse bacterial populations. The concentration of phage particles in the mixture was carefully standardized, with each of the phages in the cocktail being adjusted to achieve a uniform concentration of 10^6^ PFU/mL.

### 2.5. DNA Extraction and Genome Sequencing

The DNA of phages was extracted using the Norgen Phage DNA isolation Kit (Norgen Biotek, ON, Canada) according to the manufacturer’s instructions. DNA concentration was confirmed by nanodrop (ThermoFisher, Waltman, MA, USA). Whole genome sequencing was performed on an Illumina MiniSeq device (San Diego, CA, USA) (2 × 150 bp paired reads) with a library generated with the Nextera Flex DNA library kit (Illumina, San Diego, CA, USA). After assembly of the raw reads using Unicycler v3.6.6 [20], closely related phages were identified with BLASTn v2.13.0 [21] using the RefSeq database. VIRIDIC (Virus Intergenomic Distance Calculator) [22] was subsequently used for taxonomic classification.

### 2.6. Morphological Analysis by Transmission Electron Microscopy

Drops of phage stock dilution (10 μL, approximately 1 × 10^8^ PFU/mL) were placed on carbon-coated copper grids; after 1 min, the excess phage suspension was removed with filter paper. Equal volumes of 1% (pH 7.0) phosphotungstic acid were added for 2 min to stain the phage particles negatively, and excess solution was removed as described above. Phages were imaged using transmission electron microscopy (TEM; Zeiss, EM900, Oberkochen, Germany), with images being captured at an 80 kV accelerating voltage.

### 2.7. In Vitro Biofilm Model

Biofilms of *C. acnes* 183, 184, 186, and GG2A were established on titanium discs in a 48-well plate on titanium alloy TAN. The discs had a diameter of 13 mm and a thickness of 1 mm (surface area 1.33 cm^2^). All disks were washed, air-dried, packed, and sterilized in an autoclave at 121 °C for 20 min. To form biofilms, sterile titanium discs were placed in a 48-well plate. Then, 100 μL of the selected bacteria suspension (OD600 = 0.1) and 900 μL of sterile BHI were added to each well. The plate was incubated for 120 h at 37 °C in a static incubator without media exchanges under anaerobic conditions. The obtained biofilm was co-incubated with an individual phage and phage cocktail (10^6^ PFU/mL) for 48 h, and colony-forming unit (CFU) and confocal laser scanning microscope (CLSM) observations were used for analysis.

### 2.8. Quantification of Bacterial Load

To quantify bacterial load in the treated and control biofilms, the discs were transferred into microcentrifuge tubes containing 1 mL of phosphate-buffered saline (PBS, OmniPur PBS Tablettes, Sigma-Aldrich, Steinheim, Germany) and sonicated in an ultrasonic water bath (Model RK 510H, Bandelin electronic GmbH & Co. KG, Berlin, Germany) for 10 min. The supernatant was removed, and the bacterial pellet was resuspended in 1 mL of PBS. This process of centrifugation and resuspension was repeated four times to thoroughly wash any remaining phage particles from the samples. The CFU counts of the resuspended fluid were evaluated by performing serial dilutions and plating 10 μL streaks onto BHI plates. Plates were anaerobically incubated at 37 °C and colonies were counted after 72 h.

### 2.9. CLSM Observation

The biofilm samples were stained with the LIVE/DEAD BacLight bacterial viability kit (Thermo Fisher, Waltham, MA, USA) according to the guidelines of the manufacturer. The images were visualized by CLSM (Zeiss, LSM800, Oberkochen, Germany). Three points were randomly selected for the examination of every sample, and the biomass of selected points was quantified by COMSTAT 2.1 software [23,24].

### 2.10. Statistical Analysis

All experiments were conducted in triplicate, with each test being repeated three times, and the results are expressed as the mean ± standard deviation. The descriptive and statistical data analysis was performed and visualized using GraphPad Prism 9 (GraphPad Software). The means were compared using a *t*-test or two-way analysis of variance across multiple groups. Normal distribution was checked using the Shapiro–Wilk test, and equality of variances was measured using the Levene test. We compared non-normally distributed data using the Mann–Whitney U test. For all tests, a *p* value < 0.05 was considered statistically significant.

## 3. Results

### 3.1. Isolation and Characterization of Phages

**Figure 1:**
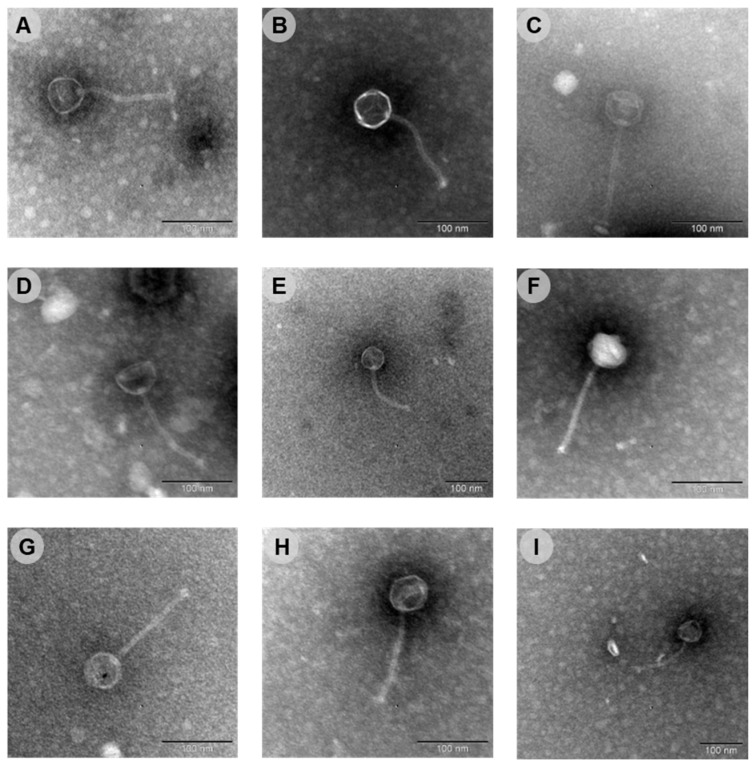
Transmission electron microscopy images of the nine isolated phages that target *C. acnes*. (**A**) CaJIE1, (**B**) CaJIE2, (**C**) CaJIE3, (**D**) CaJIE4, (**E**) CaJIE5, (**F**) CaJIE6, (**G**) CaJIE7, (**H**) CaJIE8, (**I**) CaJIE9. Each phage displays similar morphological features typical for a siphovirus. All images have a scale bar of 100 nm.

Nine phages with lytic activity towards *C. acnes* were isolated from the skin of healthy volunteers, using *C. acnes* ATCC 6919 as the indicator host. The nine phages were designated as phages CaJIE1–CaJIE9. Plaque morphology was determined after spotting each phage on a *C. acnes* ATCC 6919 bacterial lawned on a BHI agar plate and following three days of anaerobic incubation at 37 °C (Figure S1). Plaques appeared as transparent halos ranging in size from 1 to 5 mm. The morphology and dimension of all phages were also investigated by TEM analysis. The nine phages exhibited similar morphologies, with isometric heads and elongated tails (Figure 1 and Table 1).

### 3.2. Phage Genomes

The phage genomic DNA was sequenced using Illumina to further understand the relatedness of the isolated phages (Table 1). The closest related phage was identified from the NCBI RefSeq database (Table 1), revealing that the phages are all very similar to members of the Pahexavirus genus within the Caudoviricetes. This taxonomic classification was confirmed for all nine phages using a VIRIDIC analysis. None of the isolated Pahexaviruses are completely identical.

### 3.3. Host Range of the Individual Phages

**Figure 2:**
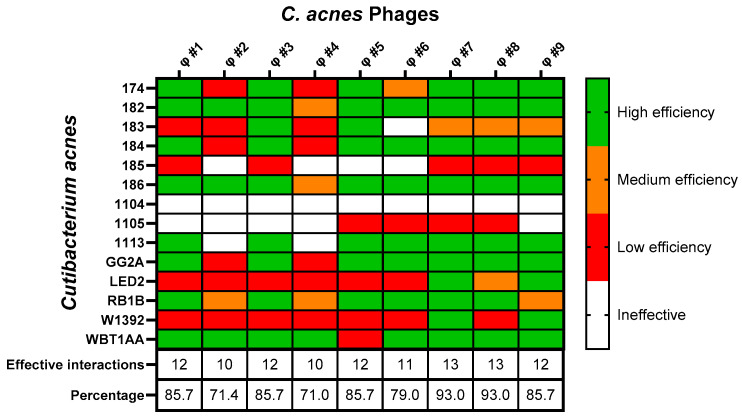
Heatmap for the host range and susceptibility of 14 clinical isolates of *C. acnes* to nine phages. The efficiency of plating (EOP) for each *C. acnes* phage against the isolates is displayed, with strains listed on the vertical axis and phages listed on the horizontal axis. EOP values, calculated by the ratio of plaques formed on test strains to those on the reference *C. acnes* ATCC 6919, are color-coded to indicate “High efficiency” (EOP ≥ 0.5, green), “Medium efficiency” (0.5 > EOP ≥ 0.2, orange), “Low efficiency” (0.2 > EOP ≥ 0.001, red), and “Ineffective” (0.001 > EOP, white). Tests were conducted in triplicate.

The infectivity of all nine *C. acnes* phages was evaluated against different clinical *C. acnes* isolates (Figure 2). Phages CaJIE7 and CaJIE8 demonstrated the broadest host range, both showing activity against 13 out of 14 bacterial strains. Phages CaJIE1, CaJIE3, CaJIE4, and CaJIE9 were effective against 12 out of 14 strains. Phages CaJIE2 and CaJIE5 had a more limited host range, both showing activity against 10 out of 14 strains. Phage CaJIE6 showed activity against 11 out of 14 strains.

Strains 174, 182, 183, 184, 186, 1113, GG2A, RB1B, and WBT1AA exhibited susceptibility to nearly all tested phages. Strains LED2 and W1392 showed intermediate susceptibility and strains 185 and 1105 showed low susceptibility. Strain 1104 was not susceptible to any of the tested phages, suggesting a potential resistance mechanism against all isolated phages.

The phages CaJIE1, CaJIE3, CaJIE7, and CaJIE8 were subsequently selected for further evaluation in a phage cocktail due to their broad host range and high effectiveness (high EOP), as they collectively target the majority of the *C. acnes* strains tested, thereby maximizing the therapeutic potential against a diverse spectrum of bacterial strains.

### 3.4. Biofilm Formation Capacity of C. acnes Isolates

**Figure 3:**
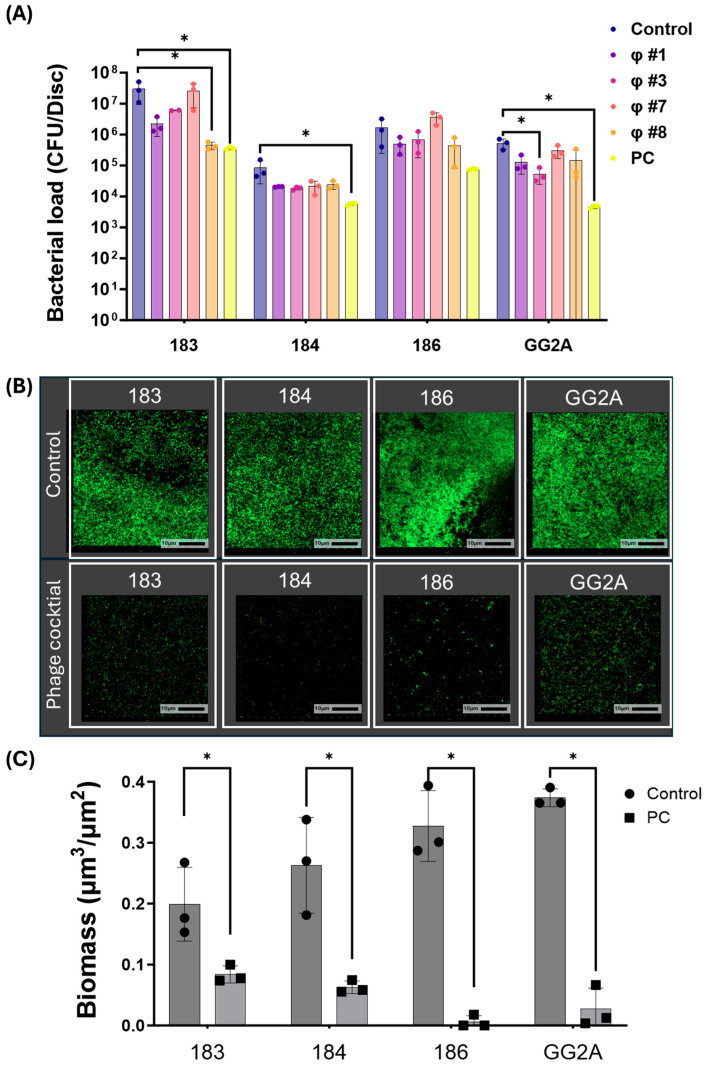
(**A**) The bacterial counts in biofilms after exposure to phage treatment. This chart compares the antibiofilm effects of a control, individual phage, and phage cocktail (PC, 10^6^ PFU/mL) on *C. acnes* biofilms for strains 183, 184, 186, and GG2A. (**B**) Confocal laser scanning microscopy (CLSM) of biofilm (strain 183, 184, 186, and GG2A) without treatment (top) and with phage cocktail treatment (bottom). The bacteria were stained with Syto9, which labels live cells in green, and propidium iodide (PI), which stains dead cells in red. The scale bars represent 10 μm. (**C**) The biofilm biomass of CLSM analysis. The chart summarizes the quantitative changes in biofilm density, where each bar corresponds to the biomass for the respective strains 183, 184, 186, and GG2A, measured 48 h post treatment with/without phage cocktail. Each dot represents individual biological replicates (n = 3 per treatment condition), with bars indicating the mean and error bars showing the standard error. Statistical significance, determined by Student’s *t*-test or a one-way ANOVA followed by Tukey post test, is indicated by asterisks: * (*p* < 0.05) indicates significant reductions in bacterial counts relative to untreated controls. Control: no treatment; PC: phage cocktail; CFU, colony forming units.

The selection of *C. acnes* strains for antibiofilm testing was based on their biofilm formation capabilities, as visualized using confocal microscopy, on medically relevant titanium substrates. Specifically, after testing 14 clinical *C. acnes* isolates, strains 183, 184, 186, and GG2A were chosen due to their comparatively greater biofilm formation, indicated by the substantial green fluorescence observed in the confocal images. The bacterial counts in these biofilms ranged from 10^5^ to 10^7^ CFU/disc (Figure 3A,B).

### 3.5. Anti-Biofilm Activity of Phages

The four bacterial strains mentioned above were used to evaluate the antibiofilm activity of individual phages and the phage cocktail. Phages CaJIE1, CaJIE3, CaJIE7, and CaJIE8 were combined to form a phage cocktail with a total final concentration of 10^6^ PFU/mL (with each phage at 2.5 × 10^5^ PFU/mL). The phage cocktail significantly reduced the CFU count for *C. acnes* strains 183, 184, and GG2A compared with untreated controls (*p* < 0.05), resulting in a 1–2.5 log_10_ reduction in bacterial numbers. For strain 183, exposure to phage CaJIE7 led to a 2 log_10_ reduction in CFU counts, while phage CaJIE3 reduced CFU counts for strain GG2A by 1.5 log_10_ compared with untreated controls (*p* < 0.05). These results demonstrate the bactericidal efficacy of both the phage cocktail and individual phages in reducing biofilm-associated bacteria (*p* < 0.05, Figure 3A).

An analysis of three-dimensional images was conducted to obtain a more comprehensive understanding of the biofilm structure. As shown at the top of Figure 3B, the CLSM images showed a compact biofilm structure in the untreated control group, whereas exposure to the phage cocktail was sparse at the bottom of Figure 3B, as evidenced by the decrease in green fluorescence after phage treatment. The quantitative analysis of fluorescence intensity demonstrated a significant reduction in biofilm biomass for all tested strains (183, 184, 186, and GG2A) following treatment with the phage cocktail compared with those of the untreated control group (*p* < 0.05, Figure 3C).

## 4. Discussion

This study investigated the potential of phage therapy as an alternative solution for treating *C. acnes* infections, particularly those associated with shoulder surgeries such as PJI. We successfully isolated nine distinct phages from the skin microbiota of healthy individuals, all belonging to the Pahexavirus within the Caudoviricetes, a taxon only containing strictly lytic *Cutibacterium acnes* phages, which are safe for phage therapy purposes [25]. Each phage demonstrated varying levels of efficacy against a range of clinical and reference strains of *C. acnes*. Among these, phages CaJIE1, CaJIE3, CaJIE7, and CaJIE8 showed the broadest host range activity and were thus further selected for the preparation of a phage cocktail. Our results indicate that the application of this phage cocktail significantly reduces *C. acnes* biofilm biomass on titanium discs, a common material used in shoulder implants, highlighting its potential therapeutic application in treating *C. acnes*-related PJI.

One of the key factors contributing to the persistence of infection in cases of PJI is the ability of bacteria to form biofilms [26]. Salar-Vidal et al. demonstrated that *C. acnes* strains isolated from healthy skin were able to produce biofilm to the same extent as isolates recovered from PJI [27]. Interestingly, a genetic analysis comparing *C. acnes* isolates from 63 patients with PJI and the skin of 56 healthy individuals revealed no statistically significant differences in the genetic profiles. These findings suggest that the *C. acnes* strains responsible for PJI likely originate from the patient’s own normal skin microbiota [28]. Importantly, phages isolated from lipid-rich areas of the skin where *C. acnes* primarily resides may also effectively target the same *C. acnes* responsible for PJI, suggesting their potential utility across different infection sites. *C. acnes* favors lipid-rich areas because it metabolizes sebum, the oily substance produced by sebaceous glands, as a primary nutrient source, which allows it to thrive in these environments [29].

Despite being isolated over different times and places, phages that target *C. acnes* show very limited genetic diversity. Liu et al. discovered that 48 sequenced *C. acnes* phages from human skin follicles shared between 85 and 100% of their genetic sequences, indicating that a single strain predominates in the skin microbiota [30]. Similarly, Marinelli et al. found that 11 isolated *C. acnes* phages lacked the diversity typical of other phages [31]. These findings align with our observations that there is limited genetic diversity among phages found on the skin, such as all sampled phages belonging to the same Pahexavirus genus. Liu et al. also found that 74 *C. acnes* strains were susceptible to the 15 tested *C. acnes* phages [30]. In our study, phages CaJIE7 and CaJIE8 showed activity against nearly all tested *C. acnes* strains. The apparent lack of genetic diversity of *C. acnes* phages and their broad host range makes them ideal candidates for phage therapy in *C. acnes*-related PJI.

The reduced efficacy of phage CaJIE7 in biofilm experiments, despite its broad activity against planktonic *C. acnes* strains (93%), may be due to the structured biofilm environment and extracellular matrix that hinder phage penetration. The slower metabolic state of biofilm-associated bacteria may limit the phage’s ability to infect and lyse these cells. The physical and metabolic differences between planktonic and biofilm cells likely contribute to this observed discrepancy. In addition, the selection of phages CaJIE1, CaJIE3, CaJIE7, and CaJIE8 for the cocktail was guided by both genomic diversity and functional efficacy. Phage CaJIE1, despite its lower efficiency of plating, was included due to its distinct genomic profile and ability to lyse strains resistant to the other phages. Phages CaJIE3, CaJIE7, and CaJIE8 were chosen for their broad host ranges, with CaJIE7 and CaJIE8 being particularly effective across the majority of clinical isolates. The slight genomic differences between CaJIE7 and CaJIE8 ensured complementary activity, providing robust coverage. The inclusion of these four phages maximized the cocktail’s efficacy by targeting a broad spectrum of *C. acnes* strains, demonstrating the value of combining phages with diverse genomic and phenotypic characteristics.

Rifampin is widely used for ODRIs due to its potent antibiofilm activity [32,33], but adjunctive rifampin therapy is not currently included in the Infectious Diseases Society of America (IDSA) recommendations for managing *C. acnes*-related PJI. Two multicenter retrospective studies reported that a rifampin combination is not markedly superior in *C. acnes*-related PJI [34,35]. This limitation brings phage therapy into focus. While not all phages can effectively degrade biofilms, certain phages have the capability to enzymatically disrupt biofilm matrices, exposing *C. acnes* cells to both phage lytic action and potentially enhanced antibiotic efficacy. The efficacy of isolated phages in this study, particularly in disrupting and penetrating biofilms, is supported by their innate ability to target specific bacterial vulnerabilities. Given these capabilities, phage therapy could be a critical adjunct or alternative to traditional methods, offering a tailored and potentially more effective treatment for *C. acnes*-related infections. Evaluating phage resistance is essential for assessing the long-term success of phage therapy, but in vitro tests fall short of replicating the dynamic conditions of a host where bacteria may evolve resistance faster. Future in vitro studies could track bacterial adaptation to phages over time or through repeated exposures to gain insights into early resistance mechanisms.

Furthermore, the limited presence of PI-stained dead cells in both the control and experimental groups can be explained by multiple factors. First, dead bacteria may have detached from the implant surface during the washing steps, resulting in their loss before staining. Additionally, the lytic activity of bacteriophages may have caused the disintegration of dead cells, preventing them from being stained by PI. The remaining PI-stained cells likely represent bacteria that were not lysed but remained attached to the surface. Moreover, the random selection of areas for CLSM analysis and the calibration of instrument thresholds could have influenced the detection of PI-stained cells in some regions of the sample.

While *C. acnes* is frequently implicated in periprosthetic joint infections, particularly in shoulder surgeries, it is important to note that up to 30% of PJIs involve polymicrobial infections, particularly in early postoperative and late chronic cases. This poses a challenge for therapeutic strategies that target only a single species, such as the phage cocktail used in this study. In the context of polymicrobial infections, a cocktail targeting multiple pathogens or the combination of phage therapy with broad-spectrum antibiotics may be necessary to achieve more effective outcomes. Future work should consider expanding the cocktail to include phages targeting other common pathogens involved in PJI to improve its clinical relevance in treating polymicrobial infections.

The primary limitation of this study is the reliance on an in vitro biofilm model to evaluate phage efficacy. While our results indicate significant biofilm reduction with the phage cocktail, a comparative analysis with the standard-of-care antibiotic, rifampicin, would provide a more robust assessment of its clinical potential. Future studies will focus on evaluating the combined effects of phage therapy and rifampicin, aiming to enhance biofilm disruption and bacterial eradication. This comparison could help establish whether phage therapy alone or in combination with rifampin might offer enhanced biofilm disruption capabilities over rifampin alone. Additionally, the observed resistance in *C. acnes* strain 1104 highlights potential limitations in the scope of phage therapy, as not all bacterial strains are susceptible to the phages tested. This resistance suggests variability in phage effectiveness and the necessity for a broader range of phages to ensure comprehensive coverage against all clinically relevant strains. One limitation of this study is that the potential interactions between phages in the cocktail were not assessed. Phages can affect each other’s characteristics, such as burst size, adsorption rate, and overall lytic activity, when used in combination. Phage–Phage interference could reduce the effectiveness of the cocktail if certain phages compete for the same bacterial receptors or inhibit each other’s activity.

## 5. Conclusions

This study validates the potential of phage therapy in treating ODRIs caused by *C. acnes*. Our phage cocktail demonstrated significant reductions in bacterial load and disrupted biofilms on prosthetic materials. These results encourage further exploration into phage therapy as a viable treatment option for biofilm-associated infections, particularly those resistant to conventional treatments. Future research should focus on optimizing phage formulations and assessing their clinical efficacy.

## Figures and Tables

**Table 1:** Morphological characteristics and genomics of CaJIE phages.

Phage	Head Length (nm)	Tail Length (nm)	First RefSeq BLASTn hit				
			Hit	Coverage	Identity	Accession	Taxonomy
CaJIE1	133.7 ± 3.6	56.4 ± 5.1	*Cutibacterium* phage PHL095N00	99%	88.68%	NC_027401.1	*Pahexavirus*
CaJIE2	129.1 ± 3.5	52.7 ± 2.9	*Cutibacterium* phage PHL171M01	97%	90.74%	NC_027346.1	*Pahexavirus*
CaJIE3	134.7 ± 8.5	51.7 ± 3.3	*Cutibacterium* phage PHL071N05	100%	91.32%	NC_022337.1	*Pahexavirus*
CaJIE4	122.3 ± 12.5	53.6 ± 2.8	*Cutibacterium* phage Lauchelly	98%	90.55%	NC_027628.1	*Pahexavirus*
CaJIE5	125.4 ± 6.4	51.2 ± 2.1	*Cutibacterium* phage P100D	99%	90.47%	NC_018852.1	*Pahexavirus*
CaJIE6	132.2 ± 1.2	52.0 ± 1.9	*Cutibacterium* phage PHL095N00	99%	88.00%	NC_027622.1	*Pahexavirus*
CaJIE7	134.8 ± 3.3	51.7 ± 1.1	*Cutibacterium* phage Wizzo	99%	91.21%	NC_027621.1	*Pahexavirus*
CaJIE8	130.5 ± 2.2	50.0 ± 2.0	*Cutibacterium* phage Pirate	99%	89.43%	NC_027623.2	*Pahexavirus*
CaJIE9	122.9 ± 1.4	50.2 ± 3.5	*Cutibacterium* phage P100_1	99%	93.42%	NC_018840.1	*Pahexavirus*

## Data Availability

All relevant data used to support the findings of this study are included within the article. Additional information and data are available from the authors upon reasonable request.

## Supplementary Materials

The following supporting information can be downloaded at: https://www.mdpi.com/article/10.3390/v16101592/s1, Figure S1: Plaque morphology of nine isolated phages that target *C. acnes* ATCC 6919. (A) CaJIE1, (B) CaJIE2, (C) CaJIE3, (D) CaJIE4, (E) CaJIE5, (F) CaJIE6, (G) CaJIE7, (H) CaJIE8, (I) CaJIE9. Scale bars represent 5 mm.
