# Supplementary material for: Isolation and Antibiofilm Activity of Bacteriophages against Cutibacterium acnes from Patients with Periprosthetic Joint Infection
Source: Viruses. 2024 Oct 10;16(10):1592. doi: 10.3390/v16101592 (PMC11512206; doi:10.3390/v16101592)
Supplement: Supplementary file 1 [file viruses-16-01592-s001.zip › viruses-3216226-supplementary.pdf]

Supplementary Materials

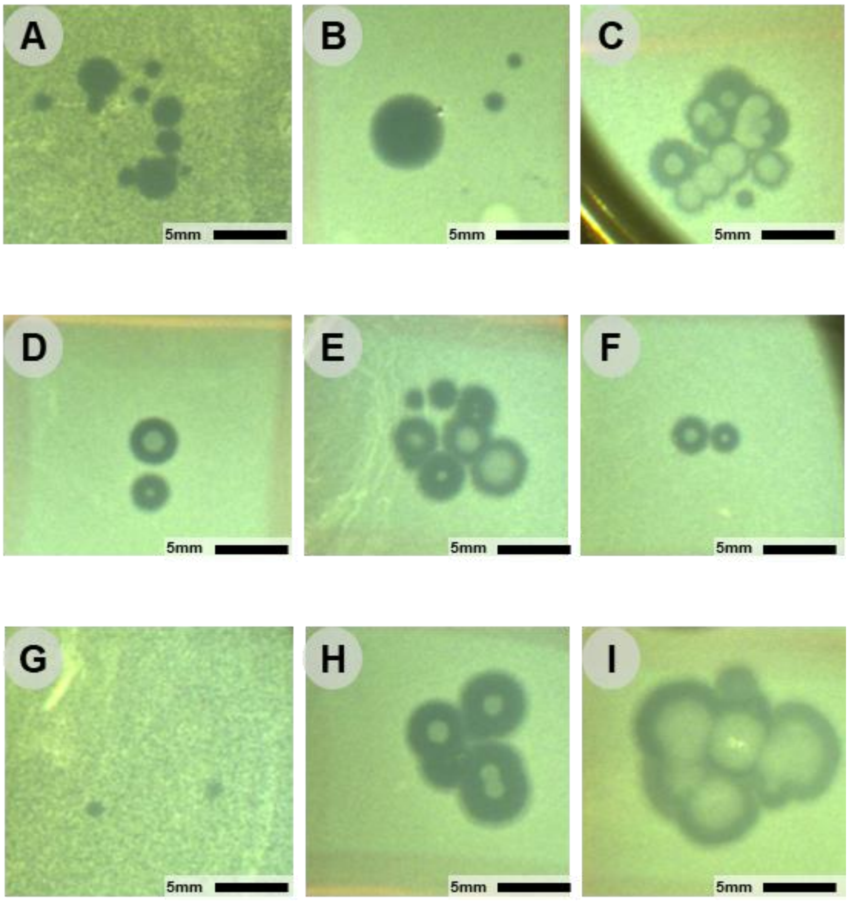

Fig. S1 Plaque morphology of nine isolated phages that target *C. acnes* ATCC 6919. (A) CaJIE1, (B) CaJIE2, (C) CaJIE3, (D) CaJIE4, (E) CaJIE5, (F) CaJIE6, (G) CaJIE7, (H) CaJIE8, (I) CaJIE9. Scale bars represent 5 mm.
